# Integrated Chinese and western medicine interventions for atopic dermatitis: a systematic review and meta-analysis

**DOI:** 10.1186/s13020-021-00506-2

**Published:** 2021-10-10

**Authors:** Chi Him Sum, Jessica Ching, Hongwei Zhang, Steven Loo, Cho Wing Lo, Mei Kwan Lai, Pui Kuan Cheong, Chau Leung Yu, Zhi-xiu Lin

**Affiliations:** 1 Hong Kong Institute of Integrative Medicine, The Chinese University of Hong Kong, Shatin, N.T., Hong Kong, China; 2 School of Chinese Medicine, Faculty of Medicine, The Chinese University of Hong Kong, Shatin, N.T., Hong Kong, China

**Keywords:** Atopic dermatitis, Integrated Chinese-Western medicine, Chinese medicine, Systematic review, Meta-analysis

## Abstract

**Background:**

Atopic dermatitis (AD) is a chronic relapsing skin disease characterized by recurring episodes of itchiness with skin erythema and surface damages. Chinese medicine (CM) is widely used for the management of AD in China not only by its own, but also used in combination with conventional therapy (integrated Chinese-Western medicine, ICWM). Although many clinical trials on the effectiveness of ICWM on AD have been conducted, however, up to date, no sound evidence has been established on the clinical effectiveness and safety of ICWM for AD.

**Objectives:**

To systematically review the currently available clinical evidence on the clinical effectiveness and safety of ICWM for AD.

**Methods:**

Randomised and quasi-randomised controlled trials, which investigated ICWM interventions with at least one control group using the same conventional interventions, no treatment or placebo treatment, were included. Four English (CENTRAL, MEDLINE, EMBASE, AMED) and three Chinese (CNKI, CBM, WanFang Med) databases were searched. Risk of bias was assessed according to the Cochrane’s tool. Meta-analysis was performed to pool the data.

**Results:**

From 1473 entries, 55 studies were included, involving 5953 participants aged between 35 days and 67 years old. Duration of treatment ranged from 1 to 24 weeks. Only 2 studies were judged to have low risk of bias, 3 studies had unclear risk of bias, and the other 50 studies were with high risk of bias. ICWM was found to be superior over WM alone in improving clinical severity of AD (measured by EASI, SCORAD), health-related quality of life (measured by CDLQI, DLQI), long term control of AD (recurrence rate), patients/investigator global score (clinical effectiveness rate), and serum IgE level. Adverse events associated with ICWM were found to be comparable with WM alone.

**Conclusion:**

ICWM seems to produce superior treatment response than WM alone in managing AD without increased risk of adverse events. However, the current available evidence remains too weak to make a conclusive decision.

## Background

Atopic Dermatitis (AD) is a chronically relapsing skin disease characterized by recurring episodes of itchiness with skin erythema, dryness, thickening and swelling [1, 2]. AD skin lesions usually appear on the face, neck, back of the hands and feet, and itchiness and sleep loss are the most significant clinical symptoms. It affects around 15% to 30% of children and 2% to 10% of adults worldwide [3, 4]. In Hong Kong, the incidence rate of AD was 15% to 20% of the general population [5].

The pathogenesis of AD involves interactions among genetic and environmental factors, skin barrier dysfunction, microbial imbalance and immune dysregulation [6–8]. Multiple factors are believed to underlie dysfunctional T-helper (Th) cell type 1, Th2, and Th17 innate and adaptive immune responses in patients with AD [7, 8]. Currently anti-inflammatory treatment with topical corticosteroids and/or topical calcineurin inhibitors is widely used for the management of mild to moderate form of AD, while systemic immunosuppressive agents, such as glucocorticoids, cyclosporine, or methotrexate, can be used in severe cases [8, 9]. Long-term topical corticosteroid use is associated with side effects including stretch marks, small red or purple spots, telangiectasia (small, dilated blood vessels on the surface of the skin), skin thinning, atrophy and acne. Topical use of excessive corticosteroids can also cause hypothalamic-pituitary axis suppression [1, 6]. The systemic immunosuppressive agents do not target specific points of immune dysregulation in AD, and may result in severe adverse events, including liver and kidney impairment. Ultraviolet phototherapy can be used for generalized AD, but it is contraindicated in children and young adults because of its long-term use is associated with the occurrence of skin cancer.

Complementary and alternative medicine has been increasingly used for the management of AD worldwide [10]. Chinese medicine (CM) is nowadays widely used for treating AD in East Asia and provides a valuable alternative treatment option. Many clinical studies have been conducted to evaluate the effectiveness of Chinese herbal medicines (CHMs) for the treatment of AD in recent decades [11, 12]. Findings suggested that oral use of CHMs may improve health-related quality of life for children with moderate or severe atopic eczema. As integrated Chinese and Western medicine (ICWM) is a common way of clinical practice in mainland China, many clinical studies have been published on the effectiveness of ICWM on AD [13]. However, currently no solid evidence is available to support the effectiveness and safety of ICWM for AD treatment.

Since 1997, the Hong Kong Special Administrative Region Government has adopted an evidence-based approach in promoting the use of ICWM. High quality evidence on the use of ICWM is in urgent need. We would therefore like to fill in the gap by conducting a systematic review focusing on the use of ICWM for AD. The evidence synthesized would likely help develop a pragmatic collaborative care model for both WM and CM practitioners for the management of AD in Hong Kong.

## Methods

### Eligibility criteria

We included randomised controlled trials (RCTs) or quasi-RCTs using a superiority design, which evaluated the use of investigated WM & CM interventions or its variants on the patients with atopic dermatitis. The herbal medicines included single herb, classical formulae, new formulae, herb-derived products and combination products. The control group should receive the same WM interventions, no treatment or placebo. The WM medicine included both oral and topical application of chemical drugs such as antihistamines, corticosteroids and other modalities such as UV light therapy.

Studies on “chronic eczema”, “subacute eczema” and “acute eczema” were generally excluded, except when they use a recognized diagnosis of AD such as the Hanifin and Rajka’s criteria or the UK working group criteria explicitly [14]. Studies on other types of eczema such as anal eczema, genital eczema, dyshidrosis, eczema rhagadiforme, keratinized eczema were all excluded. Trials with co-morbidity other than allergy-related diseases (e.g. asthma) were also excluded.

The included studies should report one or more of the following primary and secondary outcomes. The primary outcome was the clinical severity of eczema, measured by a validated or objective tool, such as eczema area and severity index (EASI), scoring atopic dermatitis (SCORAD), six area, six sign atopic dermatitis (SASSAD) severity score, investigators’ global assessment (IGA), and affected body surface area (BSA). The secondary outcomes included participant-reported symptoms, health-related quality of life, long-term control of atopic dermatitis (defined as the status of disease control at least 1 week after the end of intervention, such as recurrence rate), serum IgE level and adverse events. The percentage of trial participants with more than 50% improvement in terms of patients or investigator global score (“clinical effectiveness rate”, further explained in “Results” section) was also accepted as one of secondary outcomes as it is widely used in the studies conducted in China based on national guidelines.

Participant-reported symptoms should be measured by a validated tool, such as patient-oriented eczema measure (POEM) and Pruritus Visual Analogue Scale (pruritus VAS). Health-related quality of life should also be measured by a validated measure, such as Dermatology Life Quality Index (DLQI) and Children’s Dermatology Life Quality Index (CDLQI).

### Search strategy

Literature search strategies were developed using medical subject headings and text words related to eczema. Various synonyms of the concepts of “eczema”, “Chinese medicine”, “integrative medicine” and “randomised controlled trials” were combined by “And” to construct the searching strategies. We searched the Cochrane Central Register of Controlled Trials (CENTRAL, via Cochrane Library, searched on 16 Oct 2019), MEDLINE (via Ovid, 1948 to 16 Oct 2019), EMBASE (via Ovid, 1974 to 16 Oct 2019) and AMED (via Ovid, 1985 to 16 Oct 2019). We also searched the main Chinese databases including the China National Knowledge Infrastructure (CNKI, 1915 to 18 Oct 2019), the Chinese BioMedical Literature Database (CBM, via SinoMed, 1978 to 18 Oct 2019) and Wanfang Med Online (For dissertations and conference proceedings only, 1998 to 18 Oct 2019).

Previous systematic reviews or meta-analyses on AD were also examined to identify potential trials eligible to be included.

### Data extraction

The systematic review was conducted and reported according to the Cochrane Handbook for Systematic Review of Interventions (version 5.1) and the Preferred Reporting Items for Systematic Reviews and Meta-Analyses (PRISMA) guidelines [15, 16].

All titles and abstract of the entries returned by the search were imported into Covidence, an online collaboration tool designed to facilitate the work in different stages in systematic reviews, to remove duplicates and enable online screening. A review author (SCH) independently screened all the titles and abstracts against the original inclusion and exclusion criteria, and obtained full-texts of all entries that matched the inclusion criteria. Each of four reviewers (SCH, LMK, LCW, CPK) then screened part of the obtained full text articles and extracted characteristics of the studies. Another reviewer (LCW) rescreened the titles and abstracts classified as irrelevant to ensure completeness of inclusion, and a reviewer (SCH) rescreened the articles that did not explicitly describe atopic dermatitis (e.g. those articles that used “acute eczema”, “subacute eczema” or “chronic eczema”) to check whether AD’s criteria were actually used for those studies. All questionable cases were referred to another reviewer (LZX) for decision. The final list of included articles was then determined. We resolved disagreement through discussion, and recorded the reasons for excluding articles/studies. The review authors were not kept blind to the journal titles or to the study authors or institutions.

One review author (SCH) extracted outcome data in the included studies and input them onto an excel spreadsheet. The means, standard deviations of continuous outcomes and the numbers of events of dichotomous outcomes were recorded. Other information including age, diagnosis, type of intervention (including constituent component and dose), randomisation method, sample size, primary and secondary outcome types were also recorded.

### Risk of bias assessment

Two review authors (SCH and LCW) independently assessed potential risks of bias in all included studies using the Cochrane’s tool for assessing the risk of bias [15]. They assessed all six domains (sequence generation; allocation concealment; blinding of participants, personnel, and outcome assessors; incomplete outcome data; selective outcome reporting; and other sources of bias) in each study and assigned a scale of high, low, or unclear risk of bias. The discrepancy in judgment was resolved by another author (ZHW).

### Data synthesis and statistical analysis

We used risk ratio (RR) with 95% confidence intervals (CI) to summarize dichotomous outcome data of individual studies, and used Mantel–Haenszel random-effects model to pool the results across studies. We used the mean difference (MD) with 95% CI to summarize continuous outcome data at the end of treatment or follow-up within studies, and used the inverse-variance random-effects model to pool the results. For meta-analysis, we used random-effects model because of the expected heterogeneity of the studies. RevMan 5.1 software was used for data analyses. Forest plots were made to assess the effect size and corresponding 95% CI using random-effects models. Heterogeneity was assessed using the *I*^2^ test with the significance level set at *I*^2^ over 50% or *P* < 0.1.

We planned to perform subgroup analyses based on patients’ age (less or more than 12); however, this was eventually found to be infeasible as the majority of included studies (31 out of 56) contained patients of both age groups. We assessed the possibility of publication bias by using funnel plots when at least 10 studies reported the outcomes. Sensitivity analyses were performed to evaluate the influence of study methodological quality and that of using change from baseline data (with missing standard deviation imputed) instead of post-intervention data, when the imputation of standard deviation could be made based on available data.

## Results

### Study selection

**Fig. 1:**
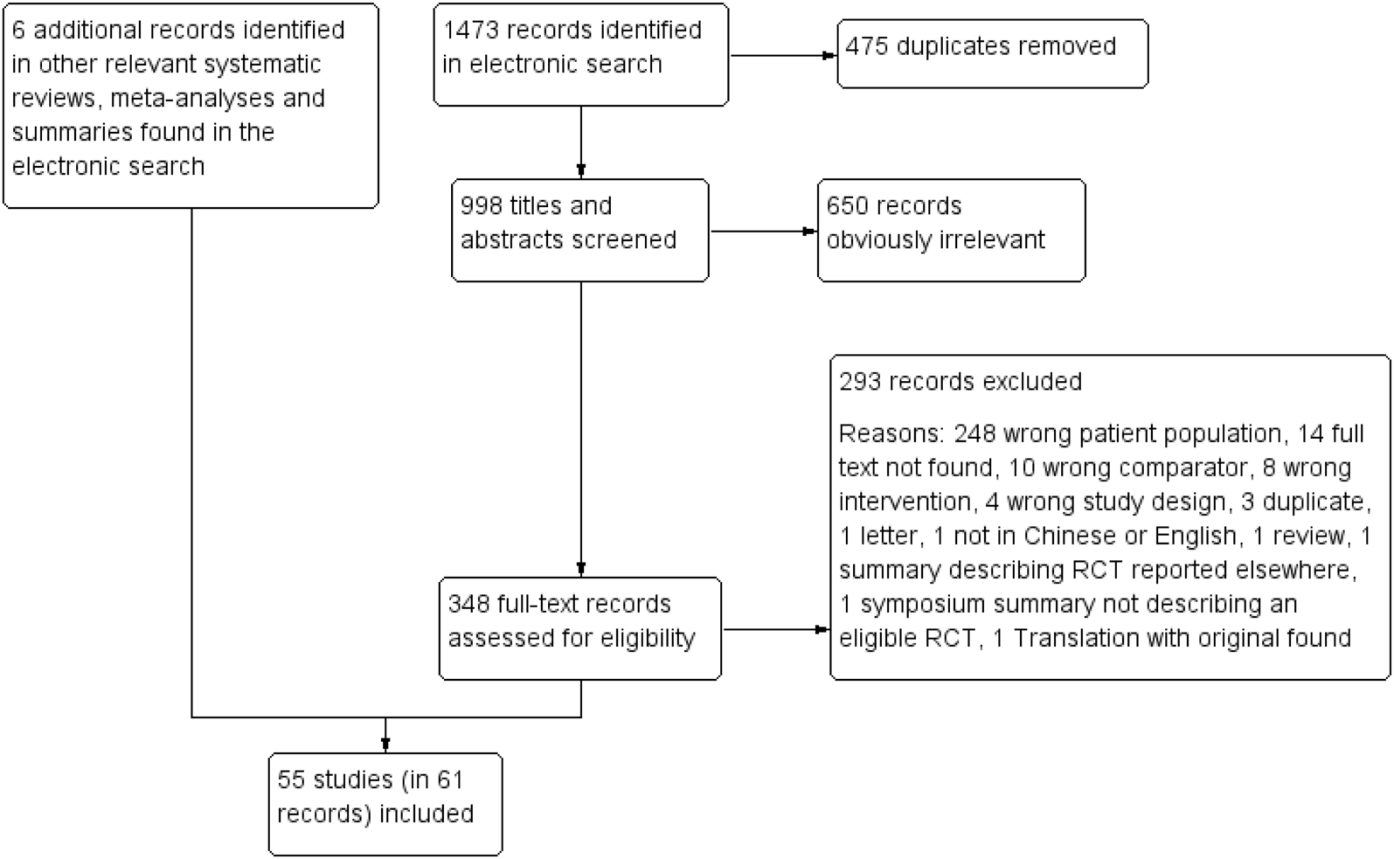
Flow of study selection

The electronic database search returned 1473 records. A total of 998 records remained after duplicates were removed. After the title and abstract screening, 650 irrelevant records were excluded and 348 full-text records were assessed for eligibility. And 6 additional records were identified from previous systematic reviews and trial summaries. Eventually, a total of 55 trials (in 61 search records [17–77]) were included. (Fig. 1, flow of study selection).

### Study characteristics

All 55 included studies were randomised controlled trials (RCTs). Among them, 5 studies were published in English and the remaining ones in Chinese. The included studies involved 5953 participants. The age of participants ranged from 35 days to 67 years old. Around 64% of the included studies (35 out of 55, involving 2987 participants) explicitly excluded patients who had received certain western medicine treatment (systemic corticosteroids: 35 studies, 2987 participants; immunosuppressants: 19 studies, 1680 participants; topical corticosteroids: 18 studies, 1256 participants; immunomodulators: 17 studies, 1577 participants; antihistamines: 16 studies, 1273 participants; antibiotics: 9 studies, 588 participants; phototherapy: 6 studies, 479 participants) within certain period (7 days to 2 months before screening) from participating in the trials.

All the included trials compared the use of Chinese herbal medicine combined with Western medicine to the use of same Western medicine alone (baseline treatment). CHMs included oral and topical use of Chinese herbal medicine in the form of decoction, granules or proprietary Chinese medicine (pCm).

Western medicine treatment included oral and topical medicines (such as emollients, antihistamines, topical corticosteroids, topical calcineurin inhibitors, urea-containing cream, antibiotics, anti-septic solutions, vitamin C or non-pharmacological therapy such as UV light therapy). The duration of treatment ranged from 1 to 24 weeks.

For primary outcomes, the included studies reported EASI, SCORAD, SASSAD, IGA (Clinical severity of eczema), POEM and pruritus VAS (Participant-reported symptoms). The most commonly reported (count ≥ 5) primary and secondary outcomes among the selected domains were clinical effectiveness rate (49 RCTs), SCORAD (20 RCTs), recurrence rate (16 RCTs), IgE level (12 RCTs), EASI (8 RCTs), and pruritus relief in Visual Analogue Scale (5 RCTs).

### Chinese medicine syndromes and Chinese herbal medicines

Twenty-seven studies mentioned some types of Chinese medicine syndrome 證候, and totally 15 syndromes were identified in those studies (Table 1). The four most common syndrome types were damp-heat 濕熱 (566 participants), spleen deficiency with dampness accumulation脾虛濕蘊 (377 participants), spleen deficiency 脾虛 (309 participants) and blood deficiency and wind-dryness 血虛風燥 (304 participants).

**Table 1:** CM syndromes identified in the included studies

CM syndromes in Chinese	English translation	No. of participants
濕熱	Damp-heat	566
脾虛濕蘊	Spleen deficiency with dampness accumulation	377
脾虛	Spleen deficiency	309
血虛風燥	Blood deficiency and wind-dryness	304
風濕熱	Wind-damp-heat	104
脾腎虛寒	Spleen and kidney yang deficiency	97
脾虛風燥	Spleen deficiency with wind-dryness	90
風濕蘊膚	Wind-dampness accumulation in skin	72
心火偏盛	Hyperactive heart fire	60
濕滯	Dampness stagnation	60
脾虛型伴有 陰虛症狀	Spleen deficiency accompanied with yin deficiency	60
脾虛心火	Spleen deficiency with heart fire	47
脾虛血燥	Spleen deficiency with blood dryness	47
胎熱型	Fetal heat	40
陰虛血燥	Yin deficiency with blood dryness	40

From the Chinese medicine perspective, the herbs used in the formulae generally have at least one of the following functions:

1. Fortifying the spleen and replenishing qi 健脾益氣 [e.g. Atractylodis Macrocephalae Rhizoma (Baizhu, 白朮), Pseudostellariae Radix (Taizishen, 太子參)];
2. Inducing diuresis to drain dampness 利水滲濕 [e.g. Coicis Semen (Yiyiren, 薏苡仁), Poria (Fuling, 茯苓)];
3. Clearing heat and drying dampness 清熱燥濕 [e.g. Phellodendri Chinensis Cortex (Huangbai, 黃柏), Scutellariae Radix (Huangqin, 黃芩)];
4. Dispersing wind and discharging heat 祛風泄熱 [e.g. Forsythiae Fructus (Lianqiao, 連翹), Dictamni Cortex (Baixianpi, 白鮮皮)];
5. Clearing heat to cool the blood 清熱涼血 [e.g. Moutan Cortex (Mudanpi, 牡丹皮), Scrophulariae Radix (Xuanshen, 玄參)];
6. Tonifying blood 養血 [e.g. Angelicae Sinensis Radix (Danggui, 當歸), Paeoniae Radix Alba (Baishao, 白芍)];
7. Nourishing yin滋陰 [e.g. Ophiopogonis Radix (Maidon, 麥冬), (Asparagi Radix (Tiandong, 天冬)];
8. Invigorating blood 活血 [e.g. Chuanxiong Rhizoma (Chuanxiong, 川芎), Persicae Semen (Taoren, 桃仁)].

The frequency of usage, functions and classifications of the top 20 most frequently used CHMs in the included studies were summarised in Table 2. These 20 CHMs can be classified into 11 categories according to their main functions in Chinese medicine. They are, in descending order of their prescribing frequency: qi-tonifying 補氣藥, heat-clearing and dampness-drying 清熱燥濕藥, water-draining and swelling-dispersing 利水消腫藥, wind-cold-dispersing 發散風寒藥, dampness-resolving 化濕藥, blood tonifying 補血藥, heat-clearing and blood-cooling 清熱涼血藥, stranguria-relieving diuretics 利尿通淋藥, qi-regulating 理氣藥, liver-pacifying 平抑肝陽藥, and heat-clearing and detoxicating 清熱解毒藥. These match well with the pathophysiology of AD in CM theory, i.e. accumulating dampness, heat and wind and the associated spleen qi deficiency that could result in blood deficiency if it persists into the chronic stage.

**Table 2:** The top 20 most frequently used Chinese medicinal herbs in the included studies

Chinese pinyin and character	Latin pharmaceutical name	Frequency of usage	Classification based on main functions (Chinese Materia Medica 7th edition)
Fuling茯苓	Poria	32	Water-draining and swelling-dispersing 利水消腫藥
Baizhu白朮	Atractylodis Macrocephalae Rhizoma	26	Qi-tonifying 補氣藥
Gancao甘草	Glycyrrhizae Radix et Rhizoma	26	Qi-tonifying 補氣藥
Baixianpi白鮮皮	Dictamni Cortex	25	Heat-clearing and dampness-drying清熱燥濕藥
Huangqi黃芪	Astragali Radix	20	Qi-tonifying 補氣藥
Cangzhu 蒼朮	Atractylodis Lancea Rhizoma	19	Dampness-resolving 化濕藥
Danggui當歸	Angelicae Sinensis Radix	18	Blood tonifying 補血藥
Yiyiren薏苡仁	Coicis Semen	17	Water-draining and swelling-dispersing 利水消腫藥
Shengdihuang生地黃	Rehmanniae Radix	16	Heat-clearing and blood-cooling 清熱涼血藥
Difuzi地膚子	Kochiae Fructus	15	Strangury-relieving diuretic 利尿通淋藥
Jingjie荊芥	Schizonepetae Herba	15	Wind-cold-dispersing 發散風寒藥
Fangfeng防風	Saposhnikoviae Radix	14	Wind-cold-dispersing 發散風寒藥
Kushen苦參	Sophorae Flavescentis Radix	14	heat-clearing and dampness-drying清熱燥濕藥
Chenpi陳皮	Citri Reticulatae Pericarpium	13	Qi-regulating 理氣藥
Huangbai黃柏	Phellodendri Chinensis Cortex	12	Heat-clearing and dampness-drying 清熱燥濕藥
Huangqin黃芩	Scutellariae Radix	12	Heat-clearing and dampness-drying 清熱燥濕藥
Jili蒺藜	Tribuli Fructus	11	Liver-pacifying 平抑肝陽藥
Dangshen黨參	Codonopsis Radix	10	Qi-tonifying 補氣藥
Zexie澤瀉	Alismatis Rhizoma	10	Water-draining and swelling-dispersing 利水消腫藥
Lianqiao連翹	Forsythiae Fructus	9	Heat-clearing and detoxicating清熱解毒藥
Shanyao 山藥	Dioscoreae Rhizoma	9	Qi-tonifying 補氣藥

A total of 42 herbal formulae with specific names were identified in the included studies. The most commonly used formulae was Danggui Yinzi 當歸飲子, a blood tonifying and wind dispersing (養血祛風) formula designed to resolve blood deficiency and wind-dryness (血虛風燥, which is a common CM syndrome in the chronic stage of AD), with 4 studies using it as the main intervention.

### Risk of bias within studies

**Fig. 2:**
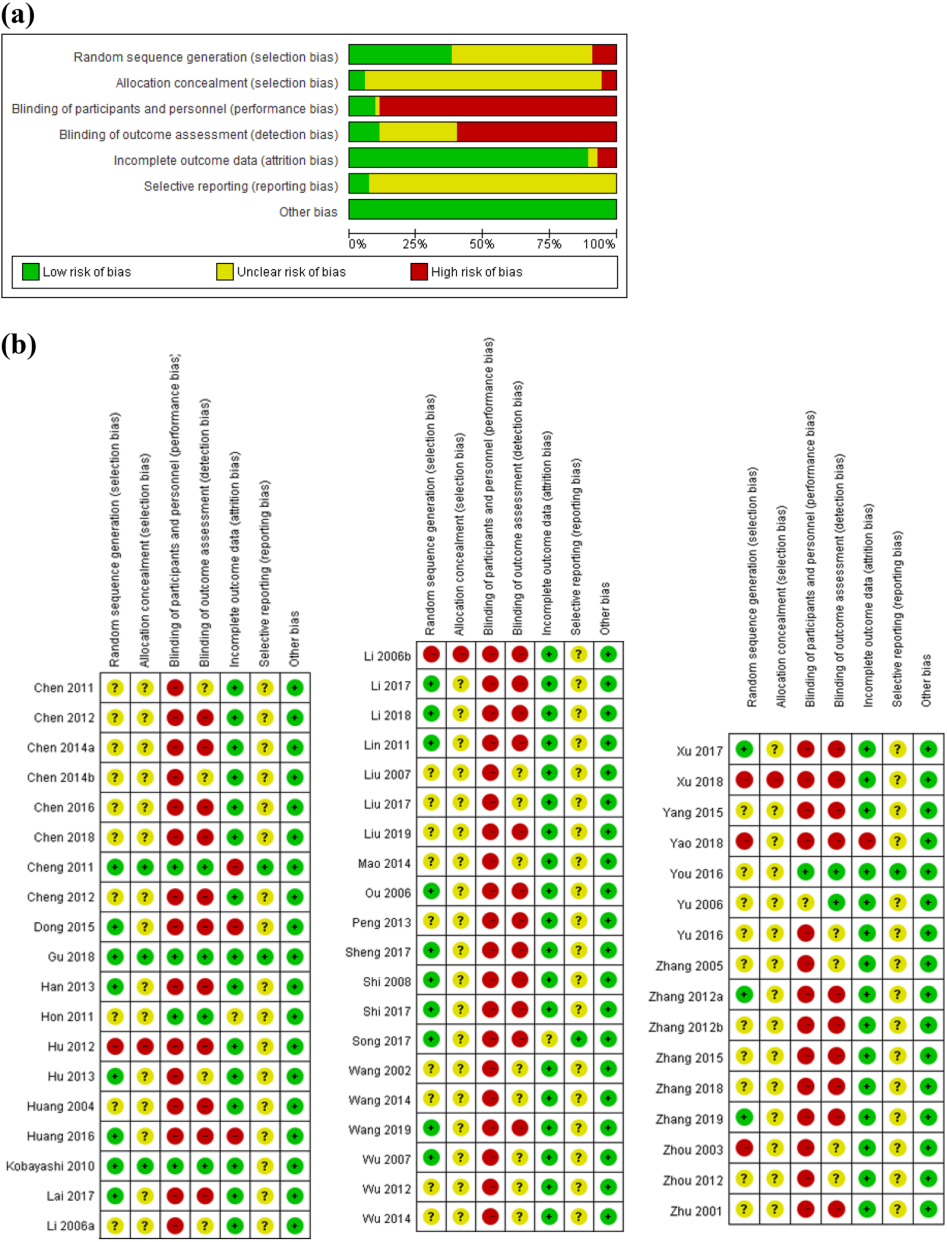
**a** Risk of bias graph; **b** risk of bias summary

About a third of included studies described an appropriate way of generating a random sequence (e.g. generation by computer software, coin tossing, random number table) and deemed to have low risk of bias in the domain of random sequence generation. Only 3 studies described the allocation concealment method, and deemed to have low risk of bias in this domain [17–19]. Five studies took measures to blind participants, study personnel and outcome assessors [17–21]. Most of the studies (47 out of 55) had low risk of bias in incomplete outcome data. Most of the studies (53 out of 55) had unclear risk of bias in selective reporting because no relevant information was available. Overall, 2 studies [17, 18] were judged to have low risk of bias, 3 studies [19–21] had unclear risk of bias, and the other 50 studies had high risk of bias. Figure 2 shows the risk of bias graphs and summaries.

### Effects of interventions

#### Primary outcomes

**Fig. 3:**
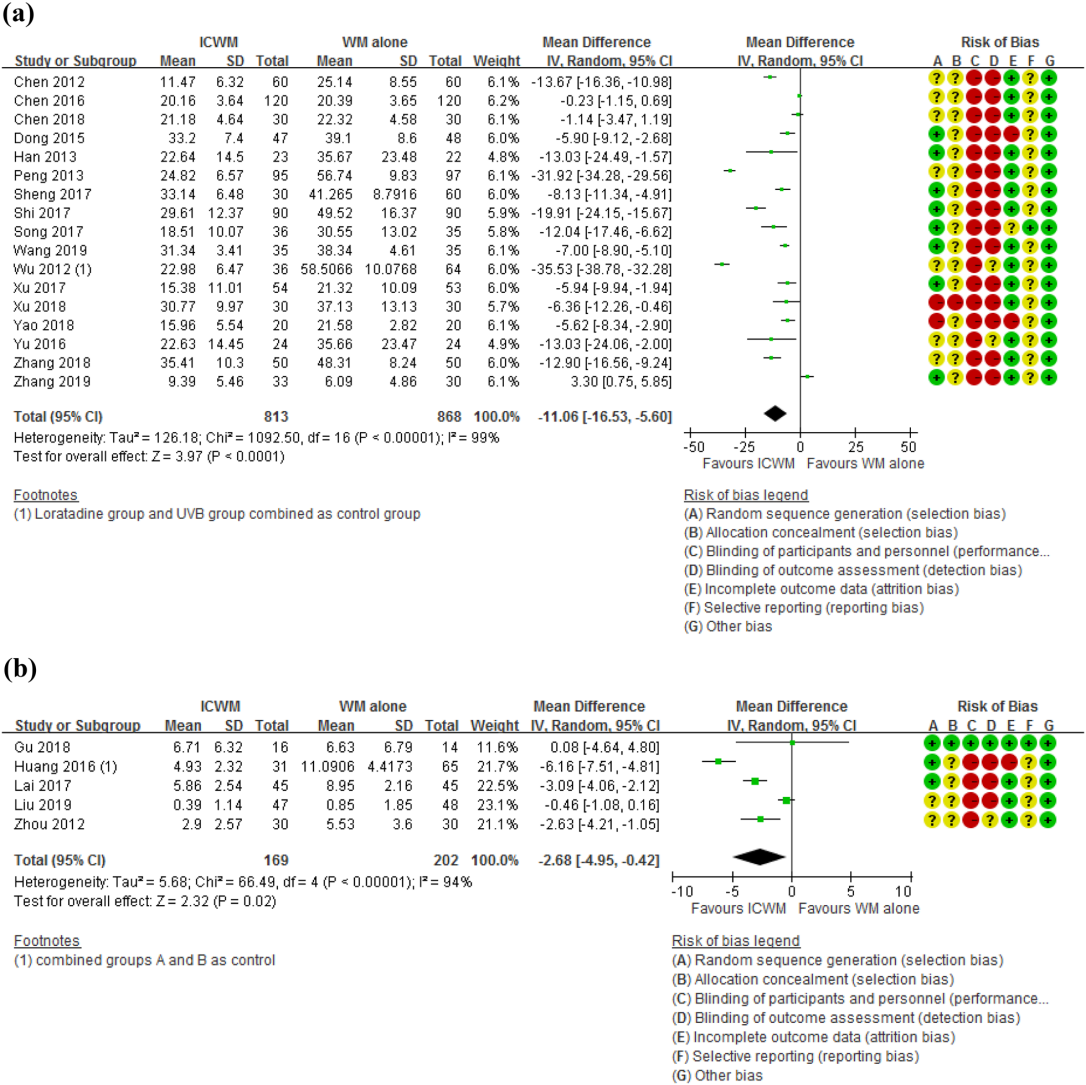
Effects of ICWM on the clinical severity of AD measured by **a** SCORAD and **b** EASI when compared with WM alone

Thirty-three studies measured clinical severity of atopic dermatitis by validated measurement scales. Among these, 18 [22, 24–29, 31, 32, 37–40, 42, 51, 54, 56, 57, 60, 68, 69] used SCORAD and 8 [17, 20, 34, 46, 55, 63, 70, 75] used EASI. Seventeen studies [22, 25–29, 31, 32, 37–40, 42, 51, 54, 56, 57, 60, 68, 69] using SCORAD and 5 [17, 34, 55, 70, 75] using EASI were included in meta-analysis because of data availability. In both measures, ICWM was superior to WM alone (SCORAD: MD = − 11.06, 95% CI − 16.53 to − 5.60, participants = 1961, I^2^ = 99%, Fig. 3a, effects of ICWM on the clinical severity of AD measured by SCORAD when compared with WM alone; EASI: MD = − 2.68, 95% CI − 4.95 to − 0.42, participants = 371, I^2^ = 94%, Fig. 3b, effects of ICWM on the clinical severity of AD measured by EASI when compared with WM alone). High heterogeneity was evidenced and possibly due to the varied interventions in these studies.

Furthermore, sensitivity analysis was conducted to examine the influence of study quality (including only study of low risk of bias) and that of using the change of score from the baseline rather than post-intervention scores as the outcome. The only study of low risk of bias [17] reported no significant difference between the ICWM and WM alone groups in the term of EASI change. When the change of score from baseline was used, with the missing SD of EASI imputed from figures reported in Gu et al. [17] and that of SCORAD imputed from Chen et al. [22], ICWM was still superior to WM alone (SCORAD: MD = − 8.15, 95% CI − 12.89 to − 3.41; EASI: MD = − 2.59, 95% CI − 4.48 to − 0.70).

Other measures included IGA [20], SASSAD [35, 36], Skin Severity Score by Japanese Dermatological Association [18] and 3 different kinds of author-defined clinical lesion/symptom scores [19, 33, 64]. Except for the Japanese study applying IGA, which showed no significant difference between ICWM and WM groups in terms of IGA (with the amount of topical agents used in the WM group substantial higher than in ICWM group), all other studies indicated that ICWM was superior to WM in their respective measures.

#### Secondary outcomes

##### Participant-reported symptoms

Six studies measured participant-reported symptoms by validated measurement scales. Among these, 5 used pruritus visual analogue scale (VAS) [20, 27, 28, 39, 46], of which 3 were included in the meta-analysis [27, 28, 39]. The result of meta-analysis showed no significant difference between the ICWM and WM alone groups (MD = − 1.21, 95% CI − 2.45 to 0.02, participants = 203, I^2^ = 96%). Only 1 study [17] measured participant-reported symptoms by POEM, and no significant difference was found between two groups.

##### Health-related quality of life

Seven studies measured health-related quality of life by validated measure scales. Among these, 3 used only CDLQI [17, 24, 55], 3 used only DLQI [23, 54, 70], and one used a score combined from both CDLQI and DLQI [31]. Two studies using only CDLQI [17, 55] and 3 studies using only DLQI were included in the meta-analysis. The study with combined score was not included for the meta-analysis as the scores were combined in an unspecified way and not separately available. In both measures, ICWM was superior to WM alone (CDLIQ: MD = − 2.12, 95% CI − 3.93 to − 0.31, participants = 125, I^2^ = 1%; DLQI: MD = − 3.12, 95% CI − 5.03 to − 1.22, participants = 206, I^2^ = 94%). However, sensitivity analysis showed no significant difference between groups in terms of CDLQI (MD = − 1.41, 95% CI − 3.84 to 1.02) when using change from baseline instead of post-intervention data, with missing SD imputed from Gu et al. [17].

##### Long-term control of atopic dermatitis (defined as the status of disease control at least 1 week after the end of intervention)

Sixteen studies measured long-term control of AD [31–33, 39, 41, 47, 50, 51, 55, 58, 60, 63, 67, 69, 70, 75]. All were expressed in some forms of “cases of recurrence” or “recurrence rate” with observing intervals from 1 week to 1 year. The pooled analysis of 16 studies showed that ICWM was superior to WM alone in reducing recurrence rate (RR = 0.47, 95% CI 0.38 to 0.58, participants = 1246, I^2^ = 0%).

##### Percentage of trial participants with more than 50% improvement in terms of patients or investigator global scores (clinical effectiveness rate)

Most included studies in Chinese (49 out of 50) reported a set of ordinal percentage measures, such as no effect rate, effective rate, significantly effective rate, or complete recovery rate, which were based on different patient’s or investigator’s global scores, either validated (like EASI, SCORAD or SASSAD) or self-defined. For each study, we grouped subjects with more than 50% improvement in terms of global scores into one category, and those lower than 50% were grouped into another. Percentages of the former categories over the total sample sizes were defined as “clinical effectiveness rates” in this review, and meta-analyzed as a dichotomous outcome.

**Fig. 4:**
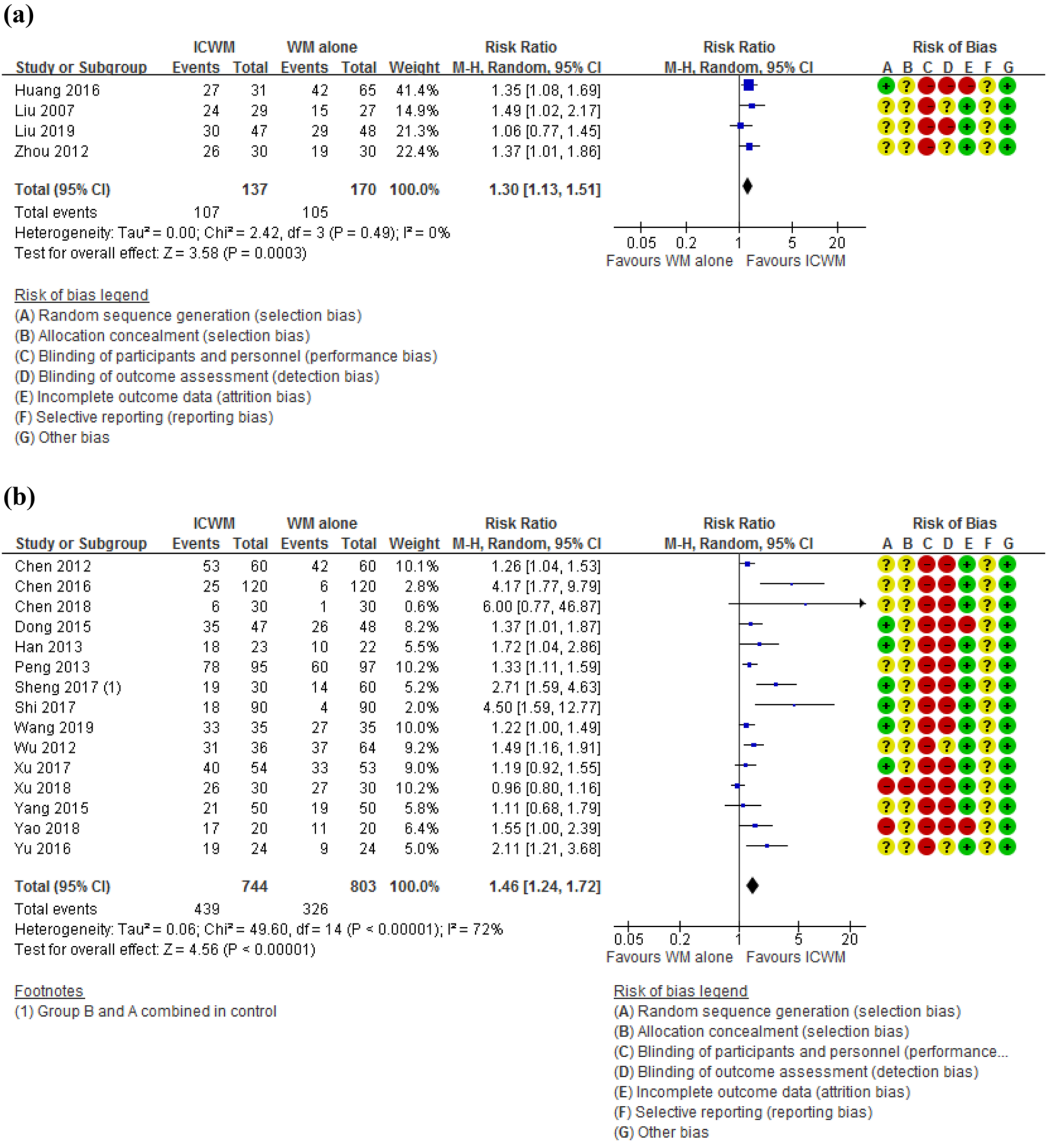

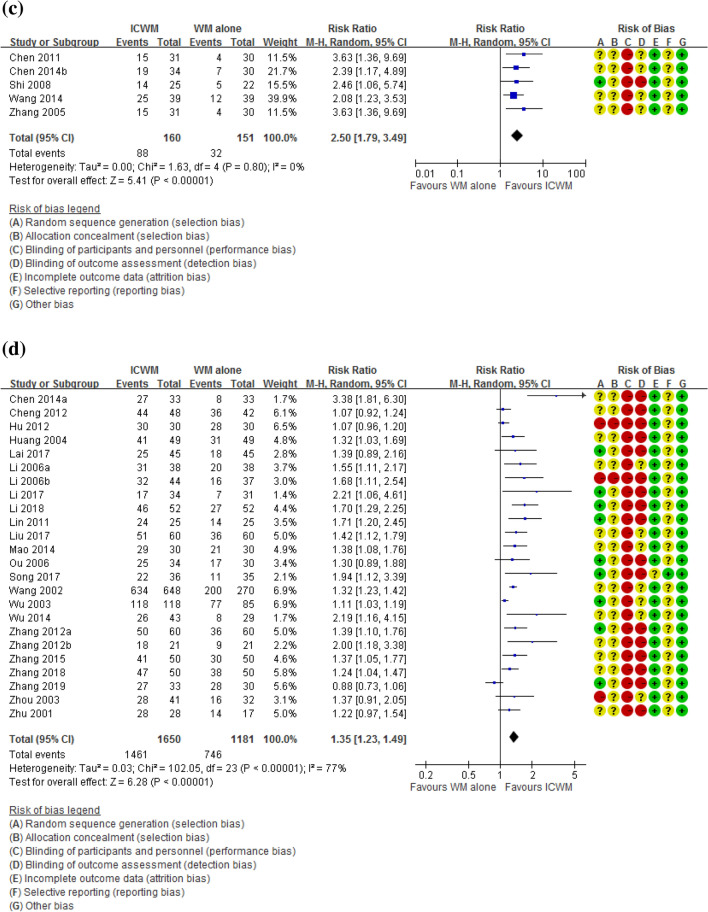
Effects of ICWM on the percentage of trial participants with more than 50% improvement in terms of **a** EASI, **b** SCORAD, **c** SASSAD and **d** all other measures when compared with WM alone

There were 4, 15, 5 and 24 studies which adopted EASI [55, 58, 70, 75], SCORAD [13, 22, 28, 29, 31, 32, 37, 38, 40, 42, 51, 54, 56, 60, 68, 69], SASSAD [30, 36, 52, 61, 62] and other measures [23, 25, 27, 33, 34, 39, 41, 44–50, 53, 59, 62, 64–67, 71, 72, 74], respectively, as their basis for calculating the percentages. Meta-analyses were performed separately for these four groups. All four groups with different measures showed that ICWM was superior to WM alone in improving clinical effectiveness rate (EASI: RR = 1.30, 95% CI 1.13 to 1.51, participants = 307, I^2^ = 0%, Fig. 4a; SCORAD: RR = 1.46, 95% CI 1.24 to 1.72, participants = 1547, I^2^ = 72%, Fig. 4b; SASSAD: RR = 2.50, 95% CI 1.79 to 3.49, participants = 311, I^2^ = 0%, Fig. 4c; all other measures: RR = 1.35, 95% CI 1.23 to 1.49, participants = 2831, I^2^ = 77%, Fig. 4d). The high heterogeneity in the SCORAD and other measurement groups was probably due to the diverse range of interventions involved.

Apart from the above, one English study [18] reported “prominent efficacy rate” (defined as the rate of subject with skin severity scoring 0 at the end of study). The difference between ICWM and WM alone groups was not significant (ICWM: 19%, 7 of 37; WM alone: 5%, 2 of 40; P = 0.06).

##### Serum IgE level

Twelve studies [19, 21, 24, 26, 30, 33, 40, 53, 58, 62, 64, 71] measured serum IgE level. Among them, 10 studies [21, 26, 30, 33, 40, 53, 58, 62, 64, 71] were included in the meta-analysis. Pooled analysis showed that ICWM was superior to WM alone in attenuating IgE level (MD = − 48.53 kU/L, 95% CI − 79.67 to − 17.38, participants = 884, I^2^ = 80%). The high heterogeneity was possibly due to the different interventions involved. For sensitivity analysis, if change from baseline data was used instead of post-intervention data, with missing SD imputed from figures report in the study Zhou et al. [64], ICWM would still be superior to WM alone in attenuating IgE level (MD = − 45.69 kU/L, 95% CI− 84.1 to − 7.29).

**Fig. 5:**
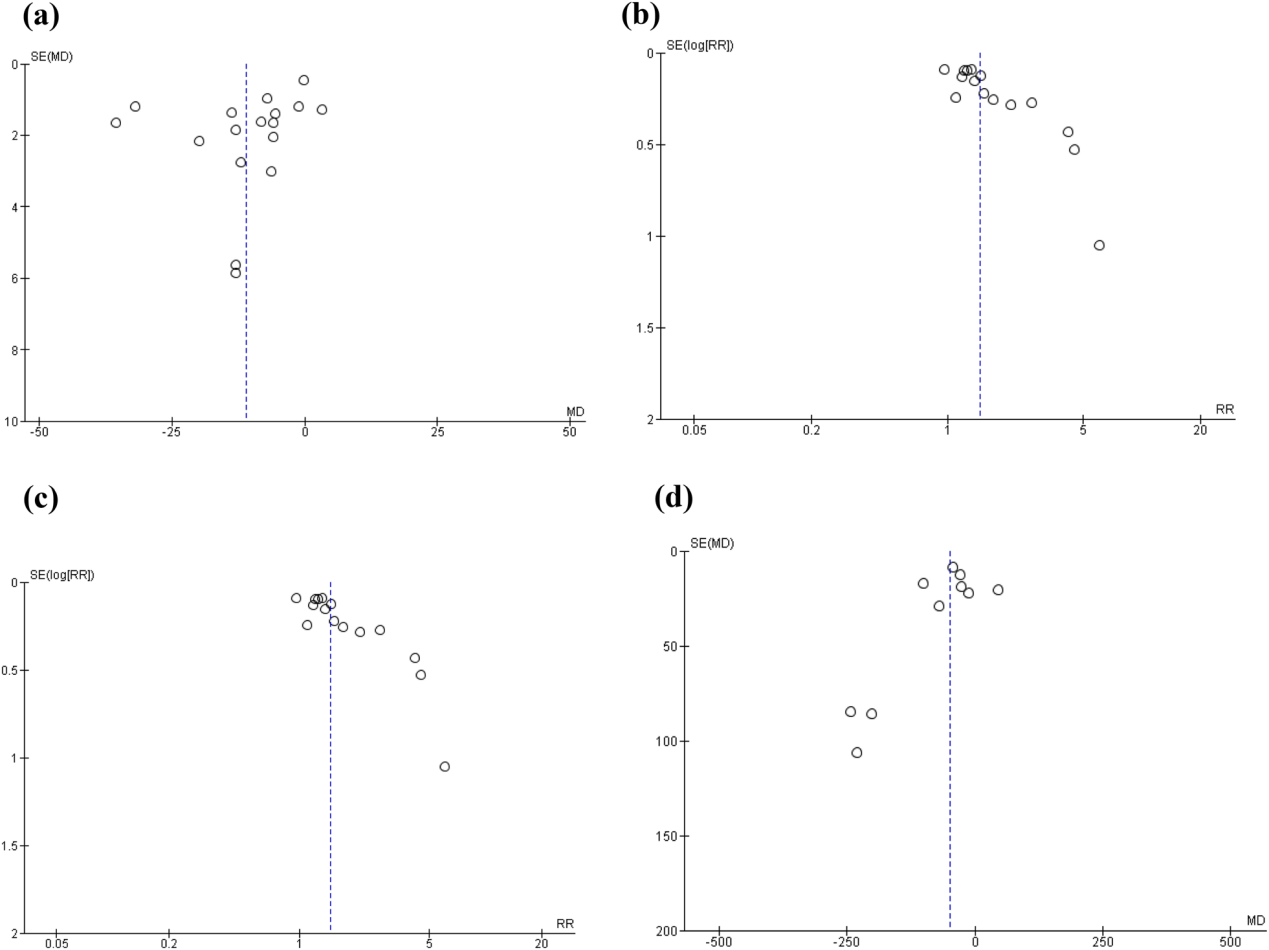
Funnel plots for the meta-analysis of **a** SCORAD, **b** percentage of trial participants with more than 50% improvement in terms of patients or investigator global score (SCORAD), **c** percentage of trial participants with more than 50% improvement in terms of patients or investigator global score (all other measures), and **d** serum IgE level

Funnel plots were made for the outcomes that have at least 10 studies included in the meta-analysis. The pooled data on SCORAD, the percentages of trial participants with more than 50% improvement in terms of SCORAD or “other measures” as defined above, and serum IgE level were examined by funnel plots. Apart from the one with SCORAD, the plots were all highly asymmetrical, suggesting a significant risk in publication bias. (Fig. 5, funnel plots).

##### Adverse events

**Fig. 6:**
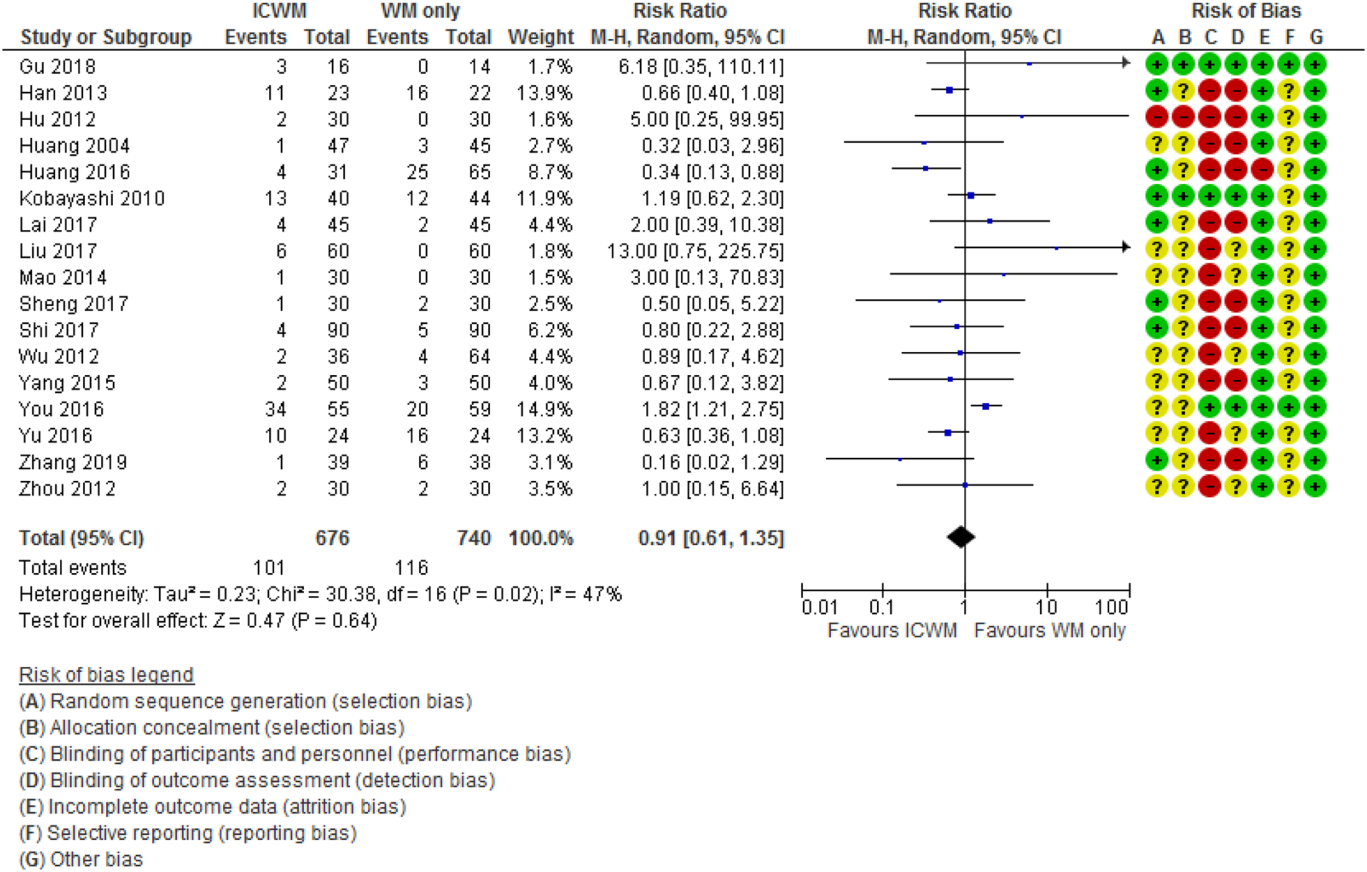
Effects of ICWM on the rate of adverse events when compared with WM alone

Thirty-five (63.6%) studies reported the occurrence of adverse events (AEs). Among these, 18 (32.7%) [19, 22–24, 28, 29, 37, 38, 41, 44, 47, 49, 53, 56, 59–61, 64, 72] studies stated that no AEs were observed. For the other 17 (30.9%) studies [17, 18, 20, 26, 27, 31, 33, 34, 40, 48, 51, 57, 66, 67, 69, 70, 75], the reported adverse events for the ICWM group mainly included skin discomfort (such as rashes, pruritus, irritation, urticaria, local edema, xerosis) and gastrointestinal disturbance (like nausea, diarrhea, stomach discomfort, abdominal distension, epigastralgia, anorexia, loose stools). Other AEs included feeling of sleepiness, insomnia, dizziness, headache, conjunctivitis, common cold, upper respiratory infection, scabies, right hypochondriac pain, malaise, rhinitis, feverish thirst, dental caries, eosinophilia, GPT elevation, IgE elevation, blood urea nitrogen (BUN) accentuation and serum potassium elevation. Pooled analysis of 19 studies found no significant difference between ICWM and WM groups in the rate of adverse events (RR = 0.91, 95% CI 0.61 to 1.35, participants = 1416, I^2^ = 47%, Fig. 6, effects of ICWM on the rate of adverse events when compared with WM alone).

## Discussion

The present research findings suggested that ICWM was superior over WM alone in improving clinical severity of AD (as measured by EASI, SCORAD), health-related quality of life (as measured by CDLQI, DLQI), long term control of AD (recurrence rate), patients/investigator global score (effectiveness rate), and serum IgE level. We found no more adverse events associated with ICWM when compared with WM alone. However, some issues related to study quality weakened the strength of evidence, such as most studies having high risk of bias, conflicting results in some sensitivity analyses and potential publication bias.

The pathogenesis of AD is a complex interplay of multiple contributing factors, such as skin barrier defects, infections, immunological factors, susceptible genes (e.g., filaggrin genes) and environmental factors (e.g., food and aeroallergens, seasonal and climatic change) [7–9]. CM also considers different influencing factors when making syndrome diagnosis of AD, albeit using different terminologies, such as damp-heat accumulation or spleen deficiency. A herbal formula is composed of herbs with different therapeutic functions to exert synergistic therapeutic actions. Experimental studies [78–82] have shown that many bioactive herbal ingredients from CHMs, including Poria (Fuling, 茯苓), Atractylodis Macrocephalae Rhizoma (Baizhu, 白朮), Angelicae Sinensis Radix (Danggui, 當歸), Atractylodis Lancea Rhizoma (Cangzhu, 蒼朮) and Dictamni Cortex (Baixianpi, 白鮮皮), exert anti-inflammatory, anti-allergic, antioxidative, and anti-angiogenic effects. These active ingredients have been demonstrated to attenuate AD through diverse mechanisms such as restoring skin barriers, balancing Th1/Th2 cells levels, as well as regulating cytokine and chemokine expression, while have few side-effects [83, 84].

The possible interaction between Chinese medicines and WM is one of the serious concerns about clinical safety. In this review, we found no significant difference in the occurrence of AEs between ICWM and WM. Our finding is congruent with previous reports that the combination of ICWM did not evoke additional adverse events [85]. It has also been reported that adding CHMs might have reduced the occurrence of adverse events of conventional pharmacotherapy such as skin dryness, skin itchiness, dry mouth/lips, and dry/scaly skin [86]. Different herbs and pharmacotherapies may be involved in the possible interaction. As CHMs can affect the pharmacokinetic properties of WMs, close attention should be paid to the possible interactions between CHMs and WMs.

To the best of our knowledge, this review is the first of its kind to systematically summarise and analyse the ICWM for AD. The comprehensive search strategy, scientifically robust way to assess risk of bias and the subsequent meta-analysis made the generation of findings more reliable. The low methodological quality and poor reporting are the main problems associated with many included studies which weakened the evidence. No proper blinding is another main methodological problem found in many included studies. As the main outcomes for evaluating the treatment effect for AD are most subjective, the blinding is a very important strategy to reduce possible bias and subjective influences. Additionally, no clear description of random generation and allocation concealment were commonly found in most of the included studies. Further clinical studies with high methodological quality are badly needed to consolidate the evidence about the ICWM for AD management.

## Conclusions

Adopting ICWM was found to be superior to using WM alone in improving clinical symptoms and quality of life, and reducing the recurrence rate in the patients with atopic dermatitis. In addition, addition of CHMs to conventional western treatment presented no more adverse effect than WM alone. However, due to the methodological limitations in the included studies and possible publication bias, we could not draw a definitive conclusion about the effectiveness and safety of ICWM for the treatment of AD. In this connection, more methodologically rigorous clinical trials are urgently needed to generate quality clinical evidence about the routine use of ICWM in the management of atopic dermatitis.

## Data Availability

Not applicable.

## Abbreviations

AD: Atopic dermatitis
ICWM: Integrated Western-Chinese medicine
WM: Western medicine
CHM: Chinese herbal medicines
RCT: Randomised controlled trials
EASI: Eczema area and severity index
SCORAD: Scoring atopic dermatitis
SASSAD: Six area, six sign atopic dermatitis
IGA: Investigators’ global assessment
BSA: Body surface area
POEM: Patient-oriented eczema measure
VAS: Visual analogue scale
DLQL: Dermatology life quality index
CDLQI: Children’s dermatology life quality index
RR: Risk ration
CI: Confidence intervals
pCm: Proprietary Chinese medicine
AEs: Adverse events
BUN: Blood urea nitrogen
